# Tissue Factor Pathway Inhibitor-2 Gene Polymorphisms Associate With Coronary Atherosclerosis in Chinese Population

**DOI:** 10.1097/MD.0000000000001675

**Published:** 2015-10-23

**Authors:** Jia Yu, Rong-Le Liu, Xin-Ping Luo, Hai-ming Shi, Duan Ma, Jun-Jie Pan, Huan-Chun Ni

**Affiliations:** From the Department of Cardiology, Huashan Hospital, Fudan University, Shanghai, 200040, China (JY, R-IL, X-PL, H-MS, J-JP, H-CN); and Key Laboratory of Molecular Medicine, Ministry of Education, Department of Biochemistry and Molecular Biology, Shanghai Medical College, Fudan University, Shanghai, 200032, China (DM).

## Abstract

Tissue factor pathway inhibitor-2 (TFPI-2) may play critical roles in the pathogenesis of atherosclerosis. In this study, we aimed to investigate the association between TFPI-2 gene polymorphisms and coronary atherosclerosis.

Four hundred and seven patients with coronary atherosclerosis and 306 individuals with normal coronary artery were enrolled in the present study. Nine single-nucleotide polymorphisms (SNPs) (rs3763473, rs59805398, rs60215632, rs59999573, rs59740167, rs34489123, rs4517, rs4264, and rs4271) were detected with polymerase chain reaction-direct sequencing method. Severity of coronary atherosclerosis was assessed by Gensini score. After the baseline investigation, patients with coronary atherosclerosis were followed up for incidence of cardiovascular events (CVEs).

Eight SNPs were in accordance with the Hardy–Weinberg equilibrium, and 8 haplotypes were constructed based on rs59999573, rs59740167, and rs34489123 after linkage disequilibrium and haplotype analysis. Two SNPs (rs59805398 and rs34489123) and 5 haplotypes correlated with coronary atherosclerosis even after adjustment by Gensini score. At follow-up (median 53 months, range 1–60 months), 85 patients experienced CVE. However, there was no strong association between the gene polymorphisms and the occurrence of CVE.

Tissue factor pathway inhibitor-2 gene polymorphisms were associated with coronary atherosclerosis in the Chinese population, suggesting that the information about TFPI-2 gene polymorphisms was useful for assessing the risk of developing coronary atherosclerosis, but there was not enough evidence showing it could predict occurrence of CVE.

## INTRODUCTION

Coronary heart disease (CHD) is the most common type of heart disease and the leading cause of death globally, resulting in over 7 million deaths, which are up from 5.2 million deaths in 1990.^1^ Therefore, it is extremely important to detect and predict the risk of CHD. Since CHD is a multifactorial disease and its molecular etiology involves the interaction of genes and environment factors and lifestyle, CHD risk assessment based on conventional risk factors cannot fully explain the risk.^2^ Atherosclerosis is considered a form of chronic inflammation resulting from interaction between modified lipoproteins, monocyte-derived macrophages, T cells, and the normal cellular elements of the arterial wall. Different kinds of cells like epithelial cell, smooth muscle, monocyte, macrophage, and platelets participate in the pathogenesis of atherosclerosis.

Tissue factor pathway inhibitor-2 (TFPI-2) could suppress endothelial cell proliferation, and promote smooth muscle cells apoptosis,^3^ suggesting that TFPI-2 may present the antiangiogenic properties,^4^ and might be an important regulator of aberrant angiogenesis.^5^ Furthermore, TFPI-2 could regulate activity of matrix metalloproteinases (MMPs).^3,6,7^ Low TFPI-2 level and the imbalance of expression of TFPI-2/MMP genes in atherosclerotic plaque contribute to imbalanced degradation and synthesis of extracellular matrix in advanced lesions, particularly in plaques with disruption.^8^ Moreover, TFPI-2 in platelets plays a role in binding to factor V and inhibits factor XI, and tissue-type plasminogen activator-induced clot fibrinolysis, regulating intrinsic coagulation and fibrinolysis.^9^ In addition, thrombin-induced macrophage TFPI-2 expression^10^ could represent a means of avoiding excessive activation of MMPs at sites of inflammation. Thrombin induced TFPI-2 expression by a mechanism involving extracellular regulated protein kinases (ERK) 1/2 and c-Jun N-terminal kinase (JNK) phosphorylation, leading finally to nuclear factor-kappa B (NF-κB) activation.^10^ What's more, TFPI-2 promotes the apoptosis of macrophages involving Fas/FasL pathway,^11^ indicating TFPI-2 might have antiatherogenic effects. Overexpression of TFPI-2 could promote atherosclerotic plaque stability in apoE^−/−^ mice.^12^

Tissue factor pathway inhibitor-2 presents a high degree of homology to TFPI-1,^13^ an important regulator of the extrinsic pathway of blood coagulation. It is a Kunitz serine protease inhibitor synthesized by endothelial cells, smooth muscle cells, and syncytiotrophoblasts, firstly known as placental protein 5. TFPI-2 exhibits inhibitory activity towards trypsin, factor XIa, plasmin, plasma kallikrein, tissue factor–factor VIIa complex, factor IX activation-polylysine, cathepsin G, and certain MMPs.^6,14–17^

Although, our previous research indicated that in early phase of acute coronary syndrome (ACS), elderly patients had low levels of TFPI-2 protein and high levels of tissue factor and MMP-1, implying that the lack of TFPI-2 may be related to ACS.^18^ However, it is still unknown whether TFPI-2 gene polymorphisms could substantially influence the risk of atherosclerosis or not. Therefore, we performed a detailed study to evaluate the association between TFPI-2 gene polymorphism and the development of coronary atherosclerosis.

## METHODS

### Study Population

During March 2008 and October 2009, 713 male and female Chinese patients who accepted coronary angiography for diagnostic purposes from the Department of Cardiology in Shanghai Huashan Hospital were recruited. Four hundred and seven patients with coronary atherosclerosis diagnosed by coronary angiography were enrolled into the case group, and 306 individuals with normal coronary angiography were enrolled into the control group.

The study was approved by the Association of Medical Ethics of the hospital. A written informed consent was obtained from all participants.

### Clinical and Biochemical Analysis

Collecting data on patients mainly include age, sex, and history of arterial hypertension, hyperlipidemia, diabetes mellitus and smoking, serum level of high-density lipoprotein (HDL)-cholesterol, and Gensini score (GSS). Serum HDL-cholesterol was measured according to standard laboratory protocols. The determination of arterial hypertension (defined as systolic blood pressure [SBP] ≥140 mm Hg or diastolic blood pressure [DBP] ≥90 mm Hg, or having been treated for hypertension) and hyperlipidemia (defined as serum low-density lipoprotein [LDL]-cholesterol ≥3.1 mmol/L or serum triglyceride [TG] ≥1.7 mmol/L) was based on AHA guideline criteria. Diabetes mellitus was diagnosed according to American Diabetes Association guideline. Medical history, current medication use, and smoking status were collected.

### Single-Nucleotide Polymorphism Selection

Single-nucleotide polymorphisms (SNPs) were selected based on HapMap data (http://hapmap.ncbi.nlm.nih.gov/) and dbSNP data (http://www.ncbi.nlm.nih.gov/projects/SNP/). The potentially functional polymorphisms were identified following the criteria: located in TFPI-2 gene promoter and exons; minor allele frequency (MAF) >0.05. Nine SNPs (rs3763473, rs59805398, rs60215632, rs59999573, rs59740167, rs34489123, rs4517, rs4264, and rs4271) were retained in our study.

### DNA Isolation and Genotyping

The target DNA segment was amplified with polymerase chain reaction (PCR) method after extraction from Ethylene Diamine Tetraacetic Acid-containing venous blood using AxypPrep DNA Blood kit (Axygen Scientific Inc., Union City, CA). The PCR products were purified using ExoSAP-IT (USB Corporation, Cleveland, OH). Sequencing products were run on 3730 XL DNA analyzer (Applied Biosystems, Foster City, CA, USA) and analyzed using software Mutation Surveyor. Data output, as fluorescent peak trace chromatograms, single peak, and double peaks, indicated homozygote and heterozygote, respectively.

### Hardy–Weinberg Equilibrium, Linkage Disequilibrium, and Haplotype Analysis

Hardy–Weinberg equilibrium (HWE), linkage disequilibrium, and haplotype analysis were tested with software SHEsis. Those SNPs in HWE were further discussed. SNPs in linkage disequilibrium could construct haplotypes. In population genetics, linkage disequilibrium is the nonrandom association of alleles at 2 or more loci that descend from single, ancestral chromosomes. The occurrence of some combinations of alleles or genetic markers is more often or less often than that expected from a random formation of haplotypes from alleles based on their frequencies.

### Coronary Angiography and Image Interpretation

Using the quantity coronary analysis (QCA) system, the severity of coronary atherosclerotic lesion was evaluated from at least 2 projections by 2 experienced cardiologists, who were blinded to the patients’ clinical information. On the basis of angiography, participants were assigned into 2 groups: control group (patients with normal angiography) and case group (patients with coronary stenosis due to atherosclerosis). The severity of coronary atherosclerosis was detected using the GSS, which is a scoring system by evaluating the degree of luminal narrowing and the geographic importance of its location.^19^ According to GSS, patients in case group were divided into 2 subgroups: low GSS group (LGS group) in which patients had low-to-middle GSS (LGS) (<40) and high GSS group (HGS group) in which patients had high GSS (≥40).^20^

### Follow-up and Outcomes

All participated patients had a routine follow-up at 6 months after inclusion, then yearly thereafter at hospital visit or by telephone or by mail. Endpoint events included ACS, stroke or transient ischemic attack, revascularization, and peripheral atherosclerosis or procedures.

The diagnosis of ACS was performed by cardiologists based on the clinical symptoms, characteristic ECG changes, cardiac enzyme levels, and the findings in coronary angiography and/or echocardiography, according to established guidelines.^21,22^ In case of fatal events, information was obtained from death certificates.

### Statistics Analysis

Statistics analyses were performed using IBM SPSS software (version 20; IBM: International Business Machines Corporation). Data were given as means and SDs for continuous variables, or as percentages for dichotomous variables. The chi-square test was used for categorical variables and the unpaired *t* test was used for continuous variables. Odds ratio (OR) with 95% confidence interval (95% CI) were used to estimate causal relations between risk factors and exposure by logistic regression analysis. Kaplan–Meier method was used to show the relationship between genotypes and occurrence of cardiovascular event (CVE). Log-rank tests were performed for comparison of genotype-dependent coronary event-free survival. Cox proportional regression was used to estimate hazard ratios (HRs) with respect to TFPI-2 genotypes for time to occurrence of CVE in case group. Two-tailed *P* values <0.05 were considered statistically significant.

## RESULTS

### Baseline Characteristics

The patients’ characteristics at baseline were shown in Table 1. The case group comprised of patients with coronary atherosclerotic stenosis, and the control group comprised of patients with normal angiography. Four hundred and seven participants in the case group were presented with higher cardiovascular risk profile when compared to the control group. They tended to be of older age, male sex, and having hyperlipidemia, lower HDL-cholesterol level, and diabetes mellitus.

**TABLE 1:**
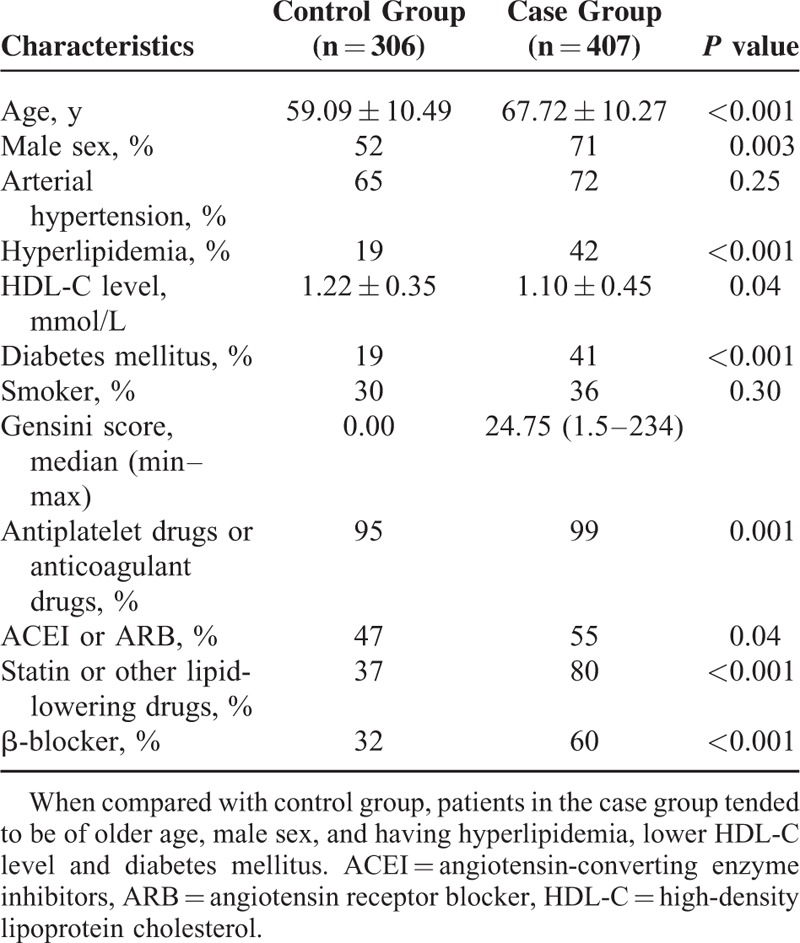
Clinical Characteristics Between Control Group and Case Group

### Genotypic Data and Hardy–Weinberg Equilibrium

**FIGURE 1:**
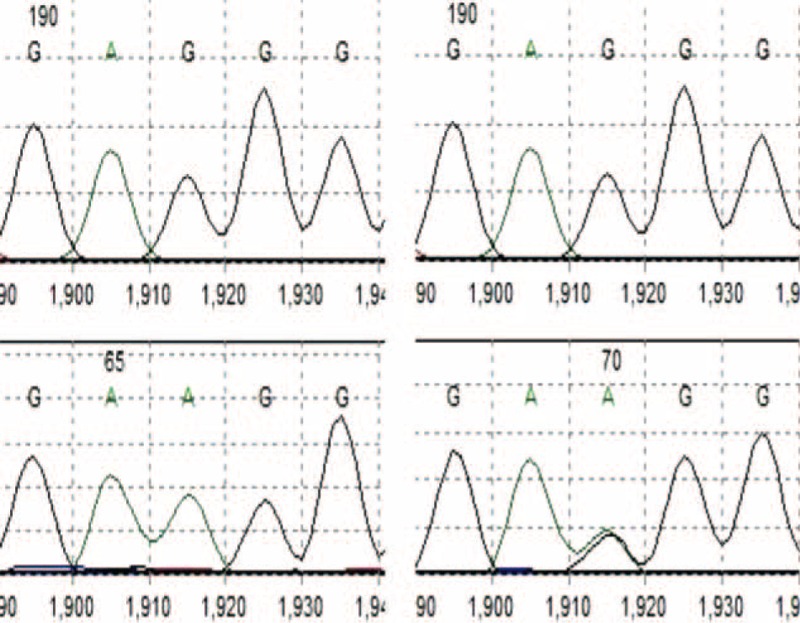
DNA sequencing and genotyping.

Alleles at different SNPs were presented in different forms in sequencing peak figures (Fig. 1). In this study, 9 SNPs were detected, which were rs3763473, rs59805398, rs60215632, rs59999573, rs59740167, rs34489123, rs4517, rs4264, and rs4264. Genotype frequencies for eight 8 complied with HWE both in the case group and the control group, except rs4271 (Table 2).

**TABLE 2:**
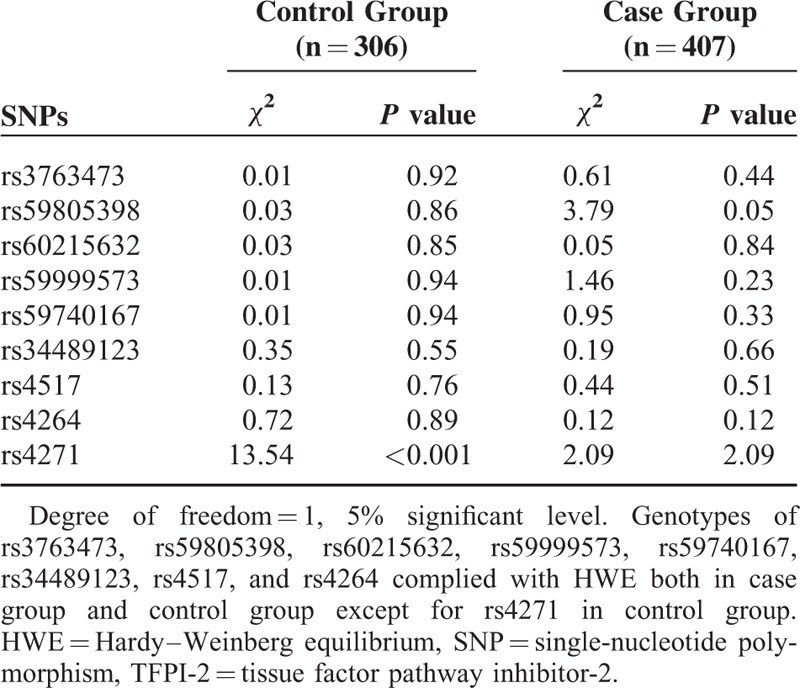
Hardy–Weinberg Equilibrium Tests of TFPI-2 Gene SNPs for Control Group and Case Group

### Two SNPs Associated with Coronary Atherosclerosis

Firstly, we evaluated the relationship between 9 SNPs and coronary atherosclerosis. As shown in Table 3, genotype distributions of rs59805398, rs34489123, and rs4271 were significantly different between the control group and the case group. Frequencies of rs59805398 CC genotype and rs34489123 AA genotype were markedly higher in the case group than that in the control group (respectively, OR 2.67 [1.82–3.91], *P* = 2.77e-7; OR 2.23 [1.14–4.39], *P* = 0.02) (Table 4). Furthermore, as compared with the control group, allele frequencies of rs59805398 C allele and rs34489123 A allele were dramatically higher in the case group (respectively, OR 1.85 [1.50–2.30], *P* = 1.27e-8; OR 1.73 [1.34–2.23], *P* = 2.34e-5). As genotype frequency of rs4271 was not in the HWE in the control group, rs4271 was excluded from relative risk calculation. After logistic regression analysis in which genotype, sex, age, hyperlipidemia, and diabetes mellitus were entered into the models, the impact of rs59805398 CC genotype and rs34489123 AA genotype on coronary atherosclerosis still existed (respectively, adjusted OR 2.88 [1.84–4.506], *P* = 3.38e-6; adjusted OR 2.76 [1.26–6.07], *P* = 0.01). After adjusted for sex, age, hyperlipidemia, and diabetes mellitus, rs59805398 C allele and rs34489123 A alleles still had a strong influence on risk of coronary atherosclerosis (respectively, adjusted OR 1.99 [1.56–2.55], *P* = 4.87e-8; adjusted OR 1.85 [1.38–2.49], *P* = 4.60e-5). These analyses revealed that there were statistically significant associations between TFPI-2 gene polymorphisms and coronary atherosclerosis.

**TABLE 3:**
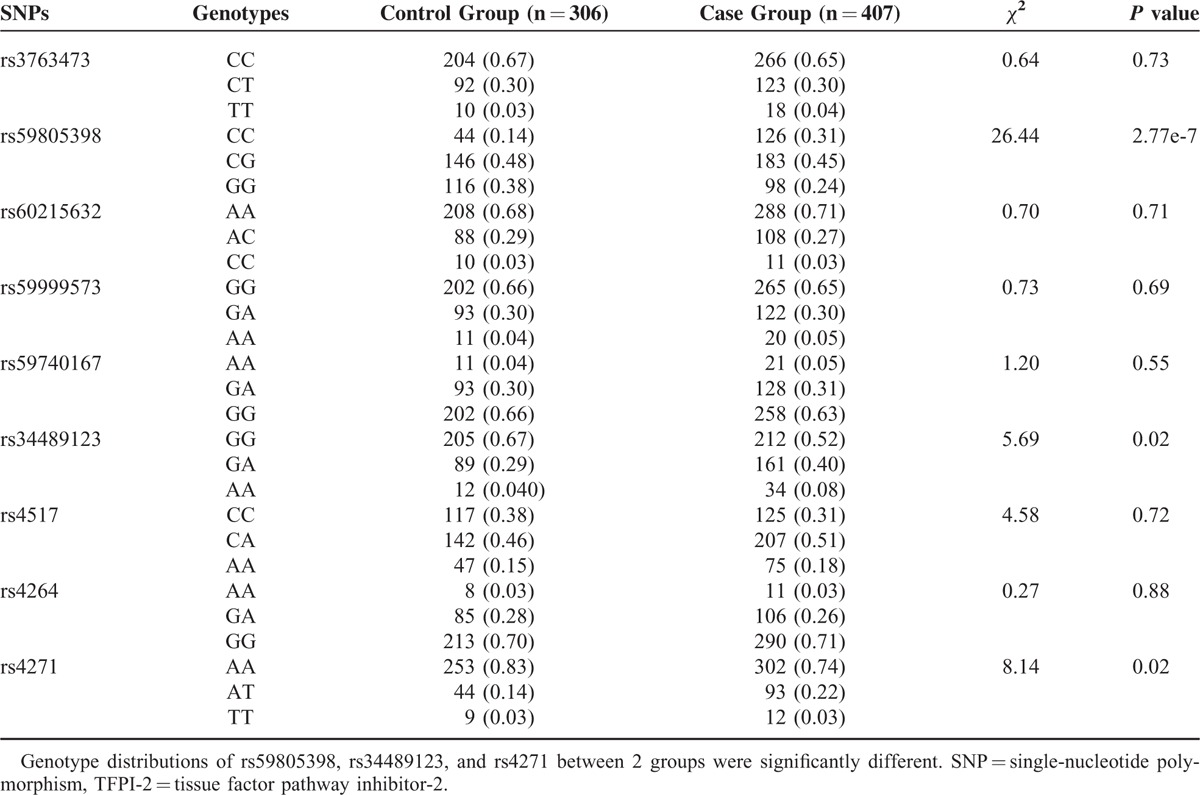
Genotype Distributions of TFPI-2 Gene SNPs between Control Group and Case Group

**TABLE 4:**
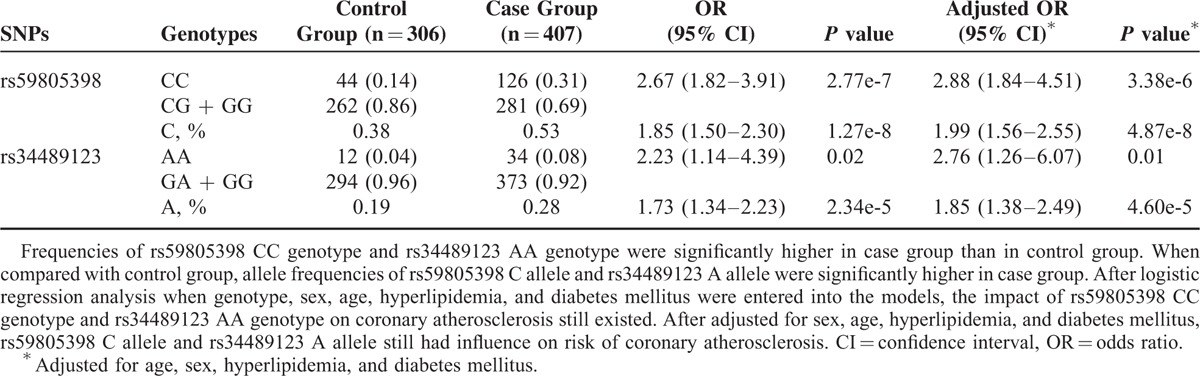
Relative Risks of rs59805398 and rs34489123 on Coronary Atherosclerosis

### Subgroup Analysis Based on Gensini Score

Participants from case group were divided into 2 subgroups based on GSS. Two hundred and nineteen patients in the LGS group had low-to-middle GSS (<40) and 188 patients in the LGS group had high GSS (≥40) (Table 5). As compared with the control group, the frequency of rs59805398 CC genotype was significantly higher both in the LGS and the HGS groups (respectively, OR 2.74 [1.79–4.20], *P* = 3.96e-6; OR 2.59 [1.66–4.05], *P* = 2.84e-5). So, it was with the frequency of rs34489123 AA genotype (respectively, OR 2.19 [1.03–4.66], *P* = 0.04; OR 2.28 [1.05–4.93], *P* = 0.04). Even when analyzing the data by multiple-factor regression analysis with adjustment for age, sex, hyperlipidemia, and diabetes mellitus, the frequencies of rs59805398 CC genotype and rs34489123 AA genotype in the LGS and the HGS groups were still significantly higher than that in the control group (respectively, adjusted OR 2.69 [1.66–4.37], adjusted *P* = 6.42e-5; adjusted OR 3.52 [1.97–6.28], adjusted *P* = 2.10e-5; adjusted OR 2.18 [1.01–4.70], adjusted *P* = 0.05; adjusted OR 4.45 [1.65–11.96], adjusted *P* = 0.003). By multiple-factor regression analysis with adjustment for age, sex, hyperlipidemia, and diabetes mellitus, the frequencies of rs59805398 C allele and rs34489123 A alleles were still significantly higher both in the LGS and the HGS groups than that in the control group (respectively, adjusted OR 1.85 [1.40–2.43], adjusted *P* = 1.36e-5; adjusted OR 2.32 [1.67–3.23], adjusted *P* = 6.04e-7; adjusted OR 1.73 [1.24–2.39], adjusted *P* = 0.001; adjusted OR 2.05 [1.39–3.02], adjusted *P* = 2.87e-4). However, the frequencies of these genotypes between the LGS and the HGS groups did not show any significant difference. This implied that these genotypes could partly predict the occurrence of coronary arthrosclerosis, but not its severity.

**TABLE 5:**
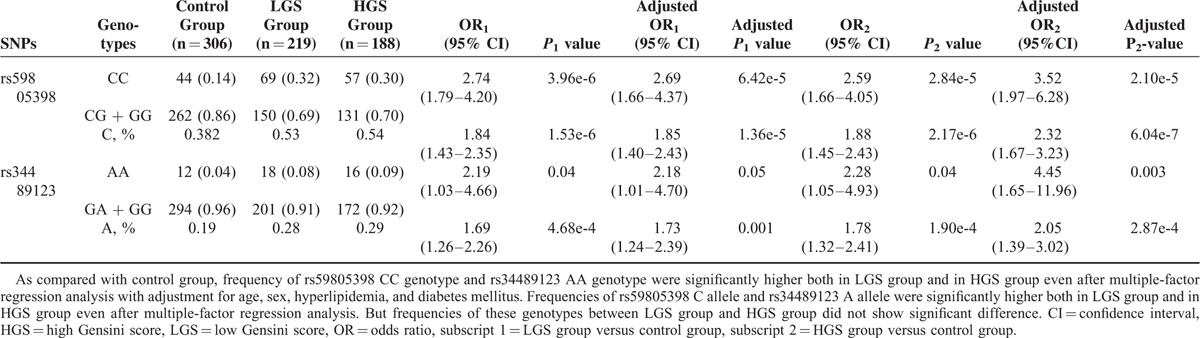
Relative Risks of rs59805398 and rs34489123 to Severity of Coronary Atherosclerosis

### Association Between Two SNPs and Occurrence of Cardiovascular Events

The median follow-up time was 53 months (range 1–60 months). Control group did not undergo CVE. The impact of TFPI-2 genes on occurrence of CVE in patients of the case group was analyzed. Eighty-five patients in the case group experienced CVE. There were 11 deaths of cardiac disease, 1 death of stroke, and 12 deaths of other causes, and mortality for patients is 5.9%.

**FIGURE 2:**
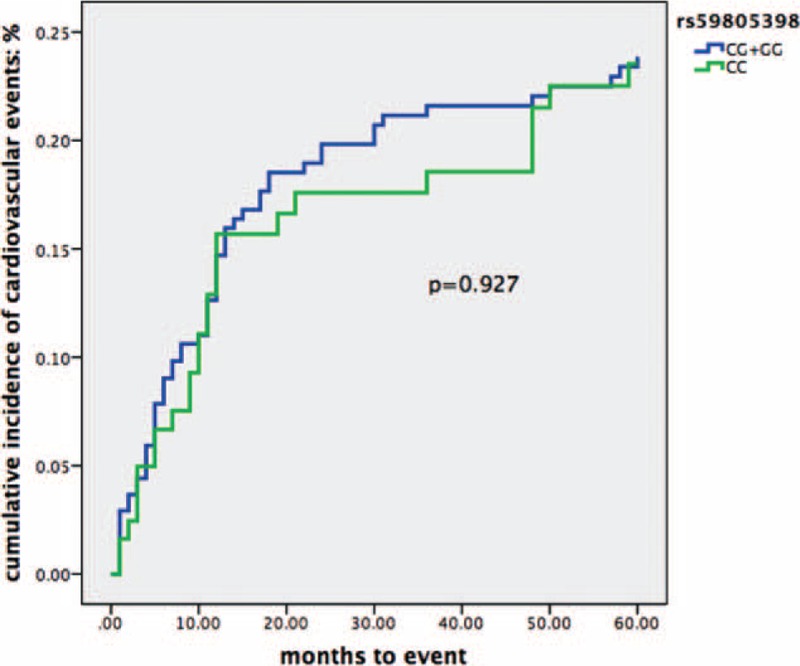
Kaplan–Meier curve of cumulative incidence of cardiovascular events in association to rs59805398. *P* value refers to log-rank test for comparison of 2 groups.

**FIGURE 3:**
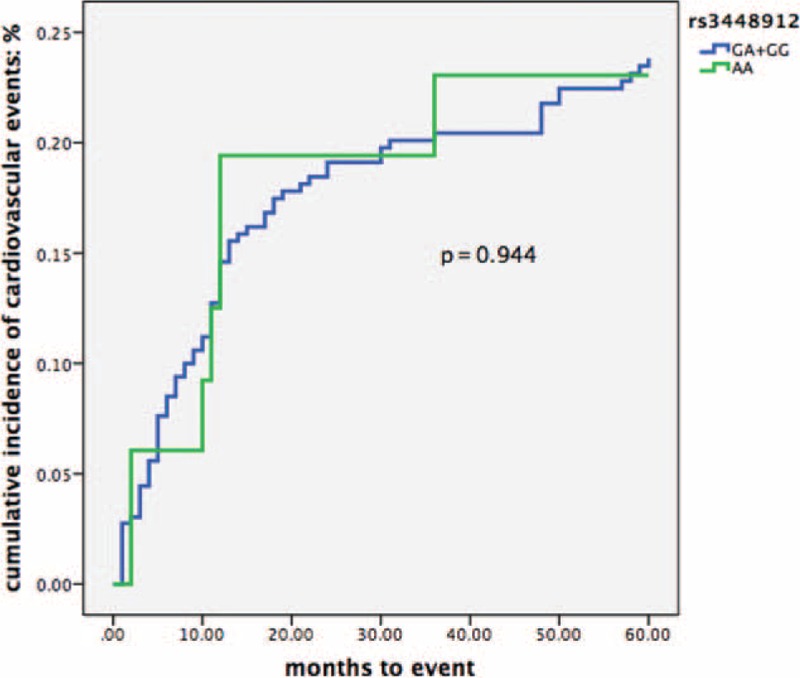
Kaplan–Meier curve of cumulative incidence of cardiovascular events in association to rs3448912. *P* value refers to log-rank test for comparison of 2 groups.

The 5-year incidence rate of CVE for rs59805398 was CC 20.8% and GC + GG 21.0% (Fig. 2). The crude HR for rs59805398 CC genotype versus GC + GG genotypes was 0.98 (95% CI 0.62–1.56, *P* = 0.93) (Table 6). Moreover, 5-year incidence rate of CVE for rs34489123 was AA 20.5% and GA + GG 21.0% (Fig. 3). The crude HR for rs34489123 AA genotype versus GA + GG genotypes was 0.99 (95% CI 0.45–2.13, *P* = 0.94) (Table 6). Taken together, the data indicated that these genotypes could not predict the incidence of CVE.

**TABLE 6:**
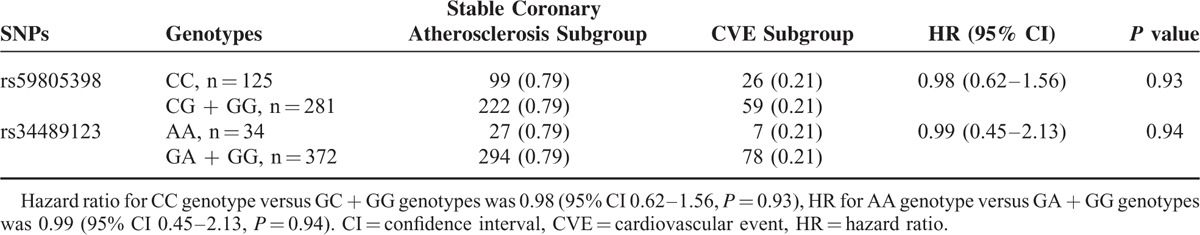
Cox Regression Analysis for the Association Between rs59805398 and rs34489123 Genotypes and Cardiovascular Events

### Association Between Haplotypes and Coronary Atherosclerosis

**FIGURE 4:**
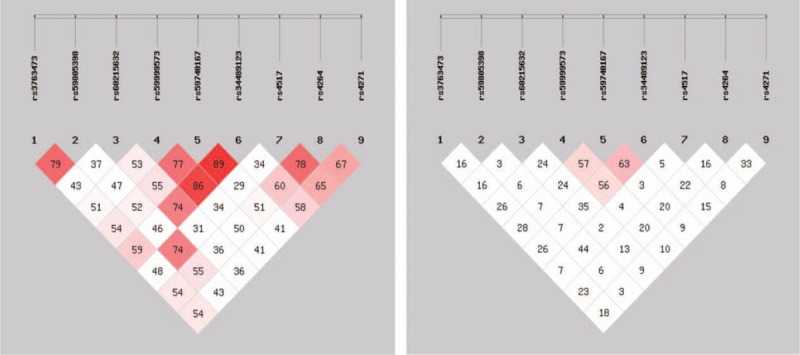
Linkage disequilibrium of TFPI-2 gene SNPs. SNP = single-nucleotide polymorphism, TFPI-2 = tissue factor pathway inhibitor-2.

Linkage disequilibrium analysis showed rs59999573, rs59740167, and rs34489123 in linkage disequilibrium (Fig. 4). Three SNPs constructed 8 haplotypes, tentatively named as Hap1 (AAA), Hap2 (AAG), Hap3 (GGA), Hap4 (GGG), Hap5 (AGA), Hap6 (AGG), Hap7 (GAA), and Hap8 (GAG).

Frequencies of these 8 haplotypes in the control or the case group were shown in Table 7. As compared with the control group, the frequencies of Hap1 (AAA) and Hap4 (GGG) in the case group were significantly lower (respectively, OR 0.69 [0.52–0.92], *P* = 0.01; OR 0.48 [0.37–0.62], *P* = 5.25e-9), whereas the frequencies of Hap3 (GGA), Hap5 (AGA), Hap6 (AGG), Hap7 (GAA), and Hap8 (GAG) were significantly higher (respectively, OR 38.29 [5.27–278.17], *P* = 2.88e-11; OR 1.04 [1.02–1.05], *P* = 8.23e-8; OR 1.02 [1.01–1.03], *P* = 4.36e-5; OR 1.06 [1.04–1.07], *P* = 2.33e-11; OR 1.02 [1.01–1.03], *P* = 9.46e-4). Even after adjustment for age, sex, hyperlipidemia, and diabetes mellitus, there were significant differences in distributions of these haplotypes between the control group and the case group. In addition, it was notable that there was no distribution of Hap5 (AGA), Hap6 (AGG), Hap7 (GAA), and Hap8 (GAG) in the control group, which implied that the patients without distribution of these haplotypes were at lower risk of coronary atherosclerosis. Taken together, these results suggested the strong relationship between these haplotypes and the occurrence of coronary atherosclerosis.

**TABLE 7:**
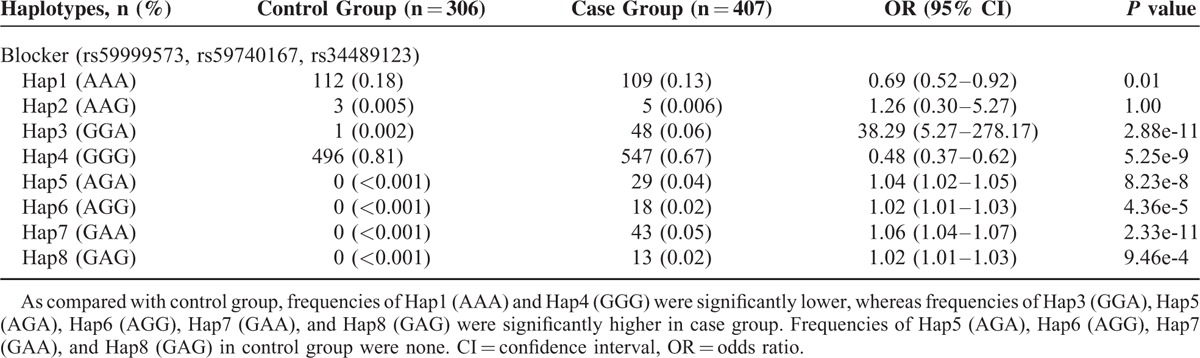
Relative Risks of 8 Haplotypes to Coronary Atherosclerosis

### Subgroup Analysis Based on Gensini Score for Haplotypes

As mentioned above, the patients of the case group were divided into 2 subgroups—LGS group and HGS group. The frequency distributions of haplotypes among the control group, the LGS group, and the HGS group were shown in Table 8. As compared with the control group, the frequencies of Hap1 (AAA) and Hap4 (GGG) were lower both in the LGS group and the HGS group (respectively, OR 0.70 [0.47–1.03], *P* = 0.07; OR 0.50 [0.32–0.77], *P* = 0.001). Furthermore, the frequency of Hap3 (GGA) was significantly higher both in the LGS group and the HGS group than that in control group (respectively, OR 30.65 [4.08–230.22], *P* = 1.97e-7; OR 53.38 [7.20–395.90], *P* = 2.28e-11). There was lack of distribution of Hap5 (AGA), Hap6 (AGG), Hap7 (GAA), and Hap8 (GAG) in the control group. In addition, the frequencies of 7 haplotypes (Hap1 [AAA], Hap3 [GGA], Hap4 [GGG], Hap5 [AGA], Hap6 [AGG], Hap7 [GAA], and Hap8 [GAG]) did not show any statistical difference between the LGS group and the HGS group. Taken together, the data implied that these haplotypes could partly predict the occurrence of coronary atherosclerosis, but not its severity.

**TABLE 8:**
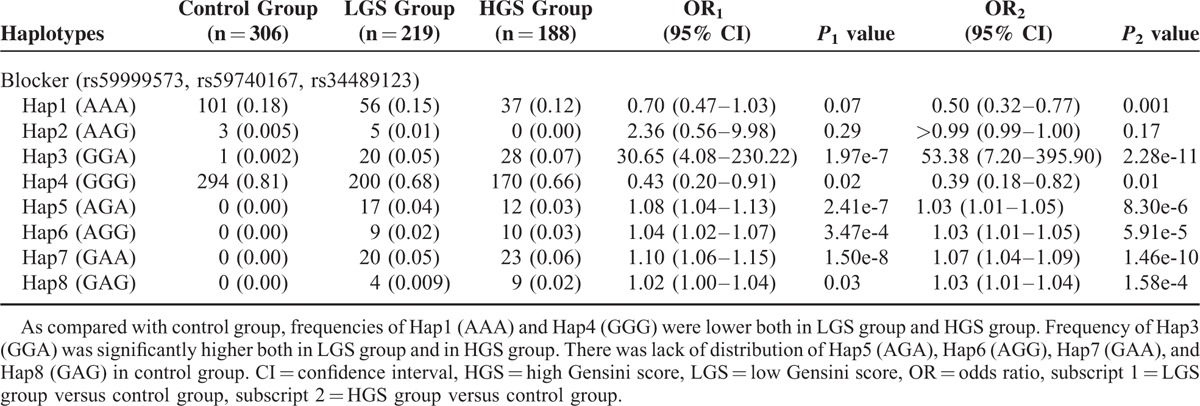
Relative Risks of Haplotypes to Severity of Coronary Atherosclerotic

### Association Between Haplotypes and Occurrence of CVE

**FIGURE 5:**
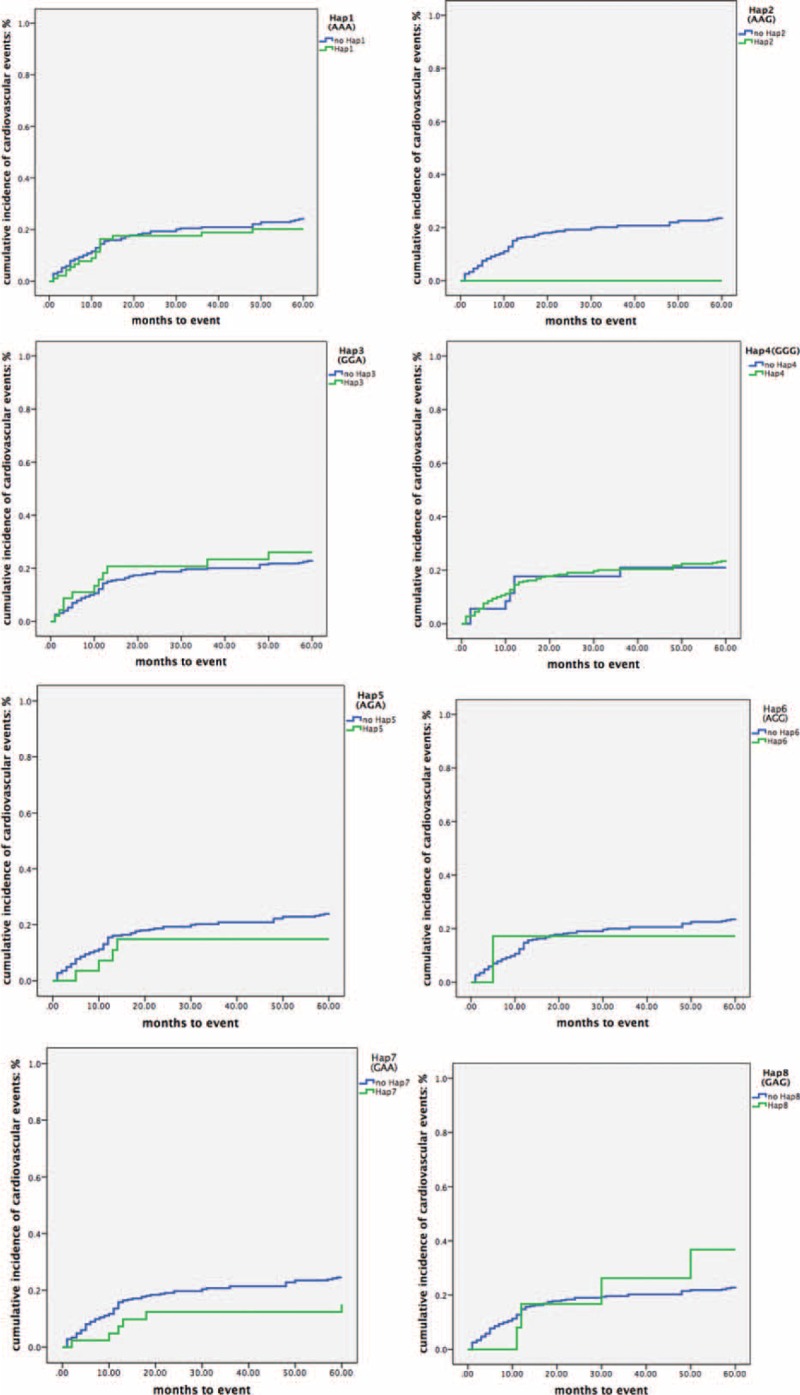
Kaplan–Meier curves of cumulative incidence of cardiovascular events in association to Hap (AAA), Hap2 (AAG), Hap3 (GGA), Hap4 (GGG), Hap5 (AGA), Hap6 (AGG), Hap7 (GAA), and Hap8 (GAG).

As mentioned above, 85 patients in the case group suffered from CVE in the follow-up period. The 5-year incidence rate of CVE for 8 haplotypes was Hap1 18.3%, Hap2 0%, Hap3 22.9%, Hap4 21.1%, Hap5 13.8%, Hap6 15.8%, Hap7 14%, and Hap8 30.8%, respectively (Fig. 5). The crude HRs of 8 haplotypes were shown in Table 9. *P* value for the 8 HRs did not reach statistical difference. This indicated that these haplotypes could not predict the incidence of CVE.

**TABLE 9:**
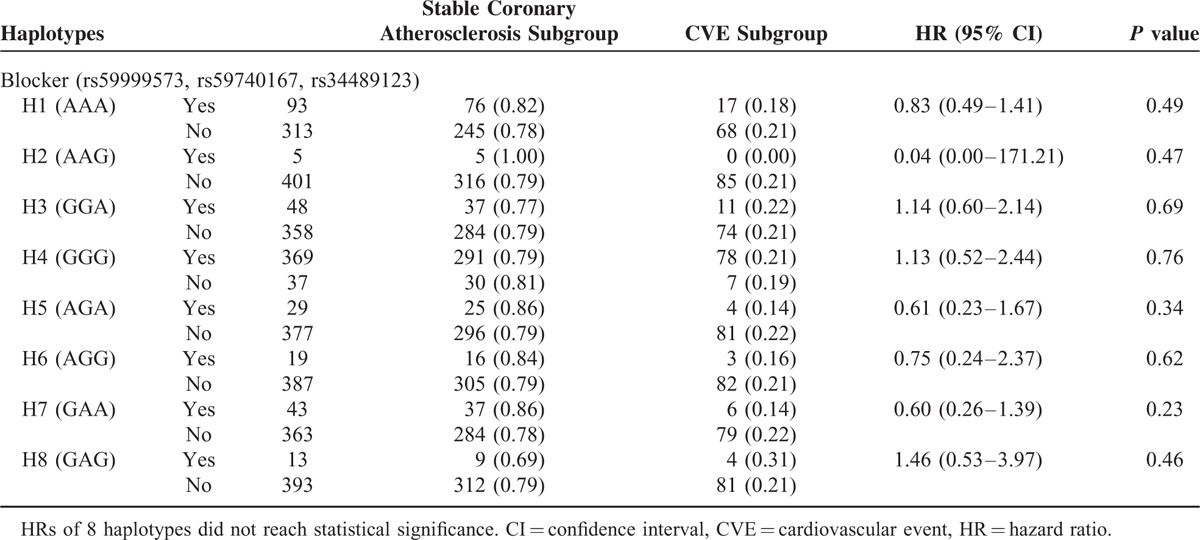
Cox Regression Analysis for the Association Between Haplotypes and Occurrence of CVE

## DISCUSSION

As a serine proteinase inhibitor, TFPI-2 associates through ionic interactions with the matrix. TFPI-2 gene is located on the human chromosome 7q22 with full length of 7 kb. The promoter of TFPI-2 gene had classic housekeeping characteristics, rich in G and C bases. GC box in the promoter had a potential transcription factor binding site and 3 transcriptional start sites, lacking of TATA box and CAAT box.^23,24^ The embryos of zebra fish depleted of TFPI-2 gene failed to undergo carcinogenesis, which suggests that TFPI-2 plays an important role in the development of heart.^25^ Many studies found that TFPI-2 could inhibit the development of atherosclerosis.^4,5,11,12^ A study performed in patients suffered from ischemic stroke showed that 2 transcription factors of TFPI-2 were changed by SNPs and that the sequence variations in transcription factor-binding sites of the promoter might influence the expression regulation of this gene.^23,26,27^ However, it has not been reported whether TFPI-2 gene polymorphism is associated with the risk of CHD.

To explore the link between TFPI-2 gene polymorphisms and coronary atherosclerosis, the detection of 6 SNPs (rs3763473, rs59805398, rs59999573, rs59740167, rs34489123, and rs4517) were executed in this study. Their influence on TFPI-2 gene function has never been reported. As was shown, rs59999573, rs59740167, and rs34489123 were in linkage disequilibrium and constructed 8 haplotypes (Hap1 [AAA], Hap2 [AAG], Hap3 [GGA], Hap4 [GGG], Hap5 [AGA], Hap6 [AGG], Hap7 [GAA], and Hap8 [GAG]). Furthermore, logistic regression analysis revealed that patients with rs59805398 CC genotype, rs34489123 AA genotype, Hap3 (GGA), Hap5 (AGA), Hap6 (AGG), Hap7 (GAA), and Hap8 (GAG) were at higher risk of developing coronary atherosclerosis, whereas patients with Hap1 (AAA) and Hap4 (GGG) were at a lower risk. Moreover, it was notable that there was no distribution of Hap5 (AGA), Hap6 (AGG), Hap7 (GAA), and Hap8 (GAG) in the control group. Thus, rs59805398 CC genotype, rs34489123 AA genotype, Hap3 (GGA), Hap5 (AGA), Hap6 (AGG), Hap7 (GAA), and Hap8 (GAG) might be genetic factors related with susceptibility to coronary atherosclerosis.

To investigate the link between TFPI-2 gene polymorphisms and the severity of coronary atherosclerosis, a subgroup analysis based on GSS was performed. The results showed that the patients with rs59805398 CC genotype, rs34489123 AA genotype, Hap3 (GGA), Hap6 (AGG), Hap7 (GAA), or Hap8 (GAG) were at higher risk of developing coronary atherosclerosis even after subanalysis based on GSS. It was strengthened that TFPI-2 gene polymorphisms were associated with coronary atherosclerosis. However, the above SNPs and haplotypes were not associated with the severity of coronary atherosclerosis, suggesting that these SNPs and haplotypes could partly predict the occurrence of coronary atherosclerosis rather than its severity.

To analyze whether the TFPI-2 gene polymorphisms were related to the occurrence of CVE, we followed up the patients in the case group for the median time of 53 months. Cox regression analysis showed that there was no statistical significance in HRs of rs59805398 CC genotype, rs34489123 AA genotype, Hap3 (GGA), Hap6 (AGG), Hap7 (GAA), and Hap8 (GAG). Thus, these SNPs and haplotypes might not predict the occurrence of CVE in a population having coronary atherosclerosis.

The mechanism for the regulation of TFPI-2 gene polymorphisms in coronary atherosclerosis is still unknown. The TFPI-2 gene promoter exhibits typical features of a housekeeping gene.

Some SNPs associated with transcription factor-binding sites of the TFPI-2 gene promoter could induce a 50% decrease in the promoter activity in vitro,^27^ indicating that sequences in the promoter probably are crucial in inducible TFPI-2 expression.^23^ The whole TFPI-2 promoter region was sequenced in the present study, but there was no SNP identified.

The 9 SNPs identified in our study are located in TFPI-2 gene introns. Most disease-causing variation of introns are located in the exon–intron boundaries, which have highly conserved sequences and obey the GT–AG rule (intron start with GT and end with AG). These boundaries are identification signal of RNA splicing. The SNPs located in the boundaries might cause the loss of exon or disturb the splicing of intron, and finally affect gene expression. Some SNPs within the introns could affect RNA splicing.^28,29^ However, the function of SNPs on genetic structure level is still unclear.

DNA methylation is one of important ways of regulating gene transcription.^30^ In several different kinds of tumors, DNA methylation in the promoter has a relationship with low level of TFPI-2.^7,31–33^ Methylation of TFPI-2 gene CpG island associated with decreased TFPI-2 expression also takes place in atherosclerotic plaques.^34^ CpG island region spans the major transcription start site and exon 1. It contains the minimal promoter and is suitable for potential DNA methylation. The role of methylation of CpG island in the progression of atherosclerosis is worthy of further study.

In summary, this is the first such study that shows TFPI-2 gene polymorphisms have an influence on developing coronary atherosclerosis. Rs59805398 CC genotype, rs34489123 AA genotype, Hap3 (GGA), Hap5 (AGA), Hap6 (AGG), Hap7 (GAA), and Hap8 (GAG) might be the vulnerable factors of developing coronary atherosclerosis. However, in patients with coronary atherosclerosis, TFPI-2 gene polymorphisms might not predict the severity of coronary atherosclerosis or the occurrence of CVE. Moreover, given the present data, there might be more research necessary to confirm our conclusion. The next direction of our research is to investigate the expression and activity of TFPI-2 of different SNPs and corresponding haplotypes, and to explore the potential ways that affect the expression and activity of TFPI-2 by SNPs, such as aberrant RNA splicing.

### Study Limitations

Several limitations of our study should be acknowledged. There were various confounders which might have occurred and influenced our results. Since diabetes, sex, age, hyperlipidemia, and diabetes mellitus are major risk factors for CHD,^35^ their effects on CHD have been considered as covariates in the logistic regression model. After adjusting for available conditions, there was still 1 important confounder that we were unable to adjust—drug use. However, it was the fact that the patients in the control group accept fewer drugs than that in the case group. What's more, there was hospitalization bias since patients enrolled were all from inpatient ward. We were able to verify only such basic conditions. In addition, the other limitations of the study include a relatively small number of patients who were enrolled to execute the genetic detection and analysis. Thus, our finding was required more researches on substantially larger sample sizes for further confirm and extrapolated to other regions and ethnic groups cautiously.
